# Unlocking multidimensional cancer therapeutics using geometric data science

**DOI:** 10.1038/s41598-023-34853-x

**Published:** 2023-05-22

**Authors:** Deepak Parashar

**Affiliations:** 1 Division of Health Sciences, Warwick Medical School, University of Warwick, Coventry, UK; 2 Warwick Cancer Research Centre, University of Warwick, Coventry, UK; 3 The Alan Turing Institute for Data Science and Artificial Intelligence, The British Library, London, UK

**Keywords:** Cancer therapy, Predictive markers, Applied mathematics, Statistics, Clinical trial design, Drug development

## Abstract

Personalised approaches to cancer therapeutics primarily involve identification of patient sub-populations most likely to benefit from targeted drugs. Such a stratification has led to plethora of designs of clinical trials that are often too complex due to the need for incorporating biomarkers and tissue types. Many statistical methods have been developed to address these issues; however, by the time such methodology is available research in cancer has moved on to new challenges and therefore in order to avoid playing catch-up it is necessary to develop new analytic tools alongside. One of the challenges facing cancer therapy is to effectively and appropriately target multiple therapies for sensitive patient population based on a panel of biomarkers across multiple cancer types, and matched future trial designs. We present novel geometric methods (mathematical theory of hypersurfaces) to visualise complex cancer therapeutics data as multidimensional, as well as geometric representation of oncology trial design space in higher dimensions. The hypersurfaces are used to describe master protocols, with application to a specific example of a basket trial design for melanoma, and thus setup a framework for further incorporating multi-omics data as multidimensional therapeutics.

## Introduction

Modern cancer therapeutics based on driver mutations observed in the DNA have shifted the objective of just finding a drug or therapy superior to the existing standard of care for all patients, to a paradigm where targeted therapies are offered to specific sub-population of patients most likely to benefit. Such a landscape of ‘*personalised medicine*’ has been plausible largely due to mutations that are not only responsible for tumour growth but also form targets for the anti-cancer drug’s mechanism of action thereby guiding treatment decisions. For example, Vemurafenib targets the BRAF mutation in advanced melanoma, Trastuzumab targets Her2 in breast cancer, and Crizotinib targeting the ALK mutation in non-small cell lung cancer, among others. Patients are screened for the presence or absence of the mutation of interest and are accordingly assigned a ‘*biomarker*’ signature. Some biomarkers track how the cancer evolves in an individual and so aid in prognosis of disease, others predict if the individual with a positive biomarker signature will respond favourably to an intervention, and some might play both the roles. These fundamental developments in cancer genomics have led to a surge in novel biomarker-guided clinical trial design approaches across the drug development pipeline^1–3^. Yet, in clinical trials as we scale up from ‘small data’ to high-dimensional biomarker data, the need to understand its shape and structure constitutes a key research gap in the literature for which appropriate visualisation techniques are necessary.

When experimental treatment options depend on one or more biomarkers within a single cancer type, several treatments may be evaluated in parallel in what is called an *umbrella trial*^4,5^. The sub-studies, which are often randomised, could be two-arm comparisons, or multi-arm comparisons of several drugs within each sub-study. New cancer trial approaches called the *basket trial* designs have been proposed to allow efficient study of a new therapy targeting a particular mutation that may be present in multiple tumour types^6–8^. In a basket trial, patients with a common mutation are recruited from populations with different tumour types and can, based on borrowing of information between tumour types, be more efficient than separate studies. The effect of experimental treatment can then be assessed both in the whole recruited group and in the individual tumour types. More complex trials in which recruited patients have different tumour types as well as different mutations are the so-called *matrix trials*^9^, that include both basket and umbrella trials as special cases.

In cancer, however, due to the large number of tumour types and mutations, the data collected at the end of a clinical study will no longer be small but potentially large and *multidimensional* and hence standard statistical techniques could be of limited use. There is ongoing effort in the community to leverage Machine Learning and Artificial Intelligence techniques to synthesize datasets and churn classification algorithms. However, these methods contribute little to our understanding of the data that comprises of multiple dimensions given that we are already dealing with multiple mutations, multiple tumour types, and multiple treatments. And, this is just the tip of the iceberg. Mutations are not the only molecular aberrations known to play a key role in prognosis and treatment prediction, there are several other realms of cancer, namely the Epigenome, Transcriptome, Proteome, and Metabolome, all of which yield rich data, and using a variety of sequencing technologies one can measure gene expression and activation, and different protein levels in cell lines enabling differentiation of blood in normal and cancer cells. It is imminent that a departure from simplistic genomic definitions of molecular targets shall involve *multi-omic* data to identify novel drug targets and hence novel therapeutic candidates^10,11^. How then does one cope with these additional dimensions when our dimension-navigation skills for matrix trials itself are somewhat limited? This motivates our goal to visualise and represent multi-omic data as multidimensional, as well as visualisation of the associated trial design space.

We take a mathematical approach to address the above problem and believe that the most intuitive way to understand the structure of multidimensional datasets is using *Geometry*. In this work, we lay the foundations of a series of forthcoming novel geometric research for cancer therapeutics by providing a geometric representation of master protocols that facilitates navigation between multiple dimensions. Our hypothesis is that the design space of a master protocol is a hypersurface (hypersphere or hypercube) with projections onto embedded lower-dimensional subspaces.

## Methods

Geometry is an ancient subject and data science isn’t new either. What is unique is their confluence. By geometry one thinks of the study of visual mathematical concepts such as the line, circle, curves etc. to prove relationships used to solve more complex mathematical problems. Geometry, however, is practical at its core as it endeavours to solve real-world problems, for example, in the field of optics, calculation of trajectories of rockets etc. and hence deeply entwined with engineering. Data science, on the other hand, is a branch of applied mathematics that deals with numerical data. These are variables that take the form of features (through refinement, selection and processing) and target variables, with the goal of finding some unknown quantity using known data in a methodical and logical manner. Such a mindset has parallels in geometry that helped people build pyramids and accomplish several architectural feats, the same that forged many modern algorithms in machine learning. Geometry, through the study of shapes, proportions and relationships holds key to help visualise certain data analytic problems that may otherwise be incomprehensible.

Indeed, geometry provides the appropriate mathematical tool to comprehend the structural features of complex multidimensional datasets^12,13^. In particular, the study of ‘topological spaces’ or ‘*manifolds*’ (mathematical term for Euclidean spaces) with embedded subspaces of lower dimensions are captured in geometric objects known as ‘*Hypersurfaces*’^14,15^. As one builds the clinical design space from the traditional setup of treatments targeting disease classifiers (2-dimensional) to treatments additionally targeting biomarkers (3-dimensional), there is a natural embedding of n-dimensional into n + 1 dimensional Euclidean space making hypersurfaces the mathematical tools of choice.

**Figure 1:**
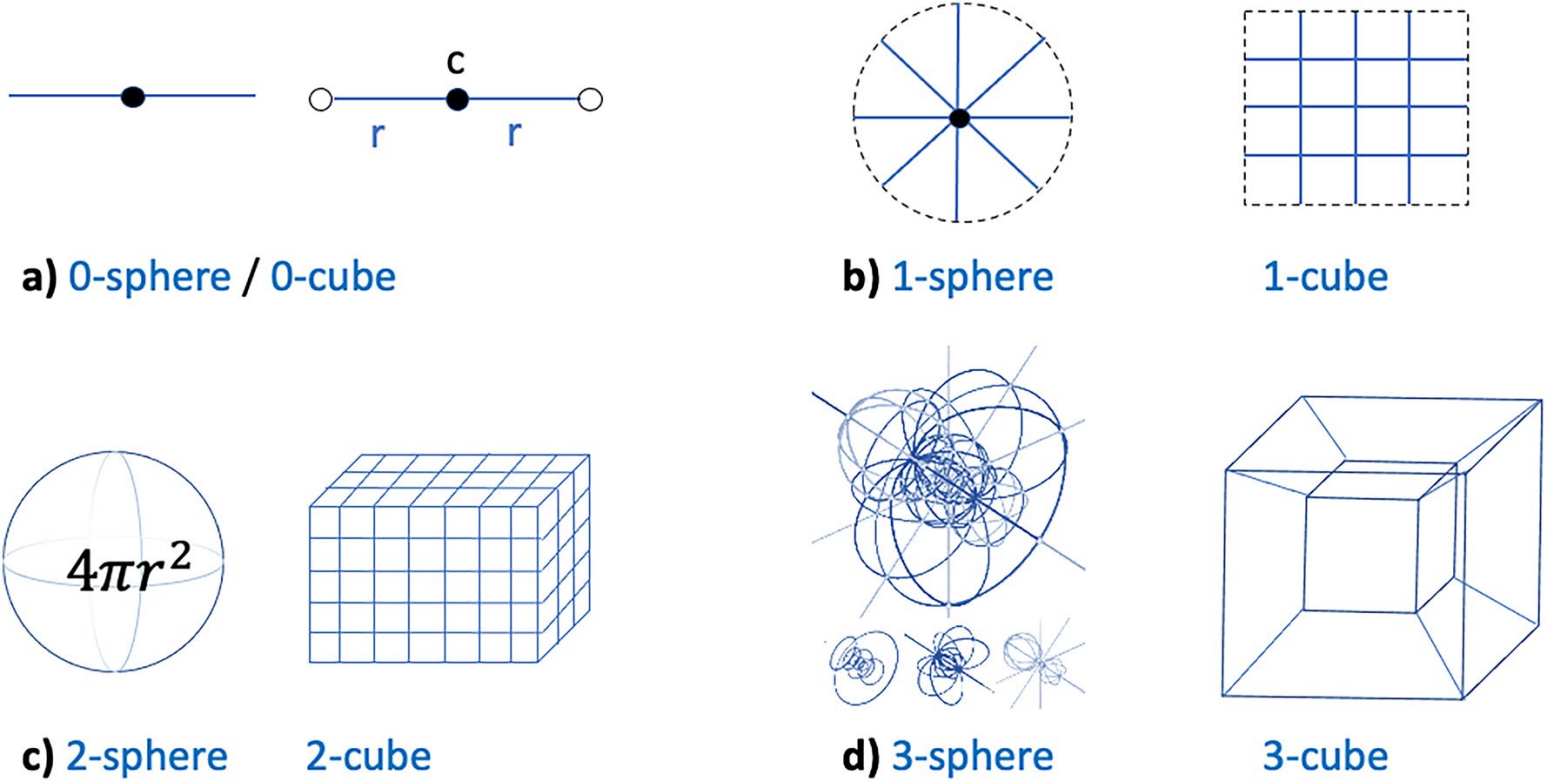
Examples of hypersurfaces. (**a**) n = 0 represents a 0-sphere or a 0-cube with boundary of the line being the pair of points {c − r, c + r}, (**b**) n = 1 represented by a 1-sphere (boundary of a disc) or 1-cube, (**c**) n = 2 is a hyperplane that can be a 2-sphere (surface of ordinary 3D sphere), or a 2-cube (net of a 3D array), and (**d**) n = 3 is a hypersurface that can be a 3-sphere (ordinary 3D sphere, not just the surface, of a 4-sphere called the Glome) or a 3-cube (ordinary cube, not just the net, of a 4-cube called the Tesseract).

Mathematically, a Hypersurface is defined as a n-dimensional manifold embedded in ambient Euclidean (n + 1) dimensional space ${\mathbb{R}}$^n+1^ of real-valued integers. Even the trivial case of n = 0 will be a 0-dimensional manifold in ${\mathbb{R}}$ (or 0-dimensional hypersurface), examples being the boundary of a line segment i.e. pair of points at either end, which may be called a 0-sphere or a 0-cube (Fig. 1a). For n = 1, i.e., 1-dimensional manifold in ${\mathbb{R}}$^2^ (or 1-dimensional hypersurface) one has y = f(x) as a single-valued function of 1 real variable. Thinking of a curve in a 2-dimensional plane, examples include the 1-sphere defined as the boundary of a disc which is a circle of certain radius; the 1-cube defined as a boundary of an array of line segments that could be a square or a rectangle (Fig. 1b). Similarly, n = 2 is a 2-dimensional manifold in ${\mathbb{R}}$^3^ (or 2-dimensional hypersurface) with y = f(x_1_,x_2_) as the more familiar hyperplane or informally the contour plot. Examples include the 2-sphere defined as the boundary of an ordinary ball i.e., surface of ordinary 3D sphere, or a 2-cube as the boundary of a 3D array of line segments i.e., a net (Fig. 1c). For n = 3, y = f(x_1_,x_2_,x_3_) i.e., a 3-dimensional manifold in ${\mathbb{R}}$^4^ (or 3-dimensional hypersurface) examples being the 3-sphere defined as boundary of a 4-ball i.e. ordinary 3D sphere or a 3-cube defined as boundary of a 4-cube i.e. an ordinary cube (Fig. 1d).

**Figure 2:**
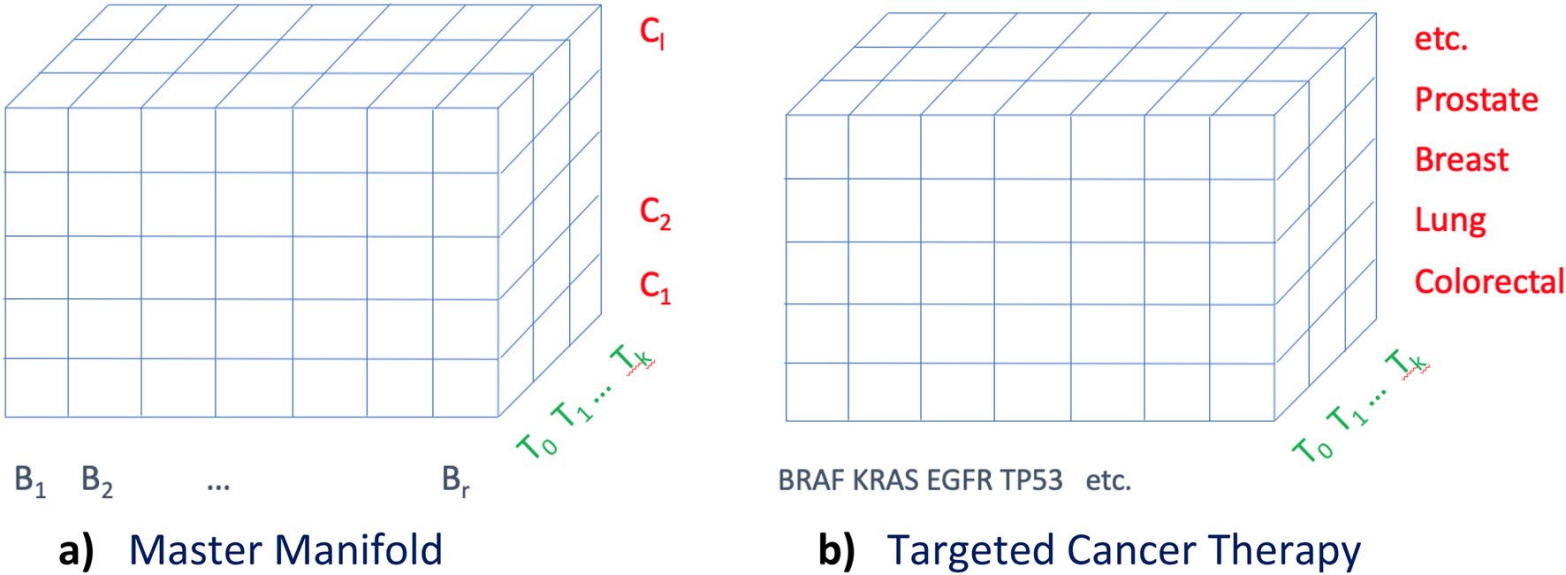
Master Manifolds. (**a**) The 3 dimensions of a master protocol for a generic disease area represented as a 3D Euclidean space, termed Master Manifold, (**b**) Master protocol for cancers represented as a Master Manifold by mapping the dimension of Disease classifiers to tumour-types and Biomarkers to DNA driver mutations.

Generically, therefore, an n-cube forms the boundary of a (n + 1)- dimensional hypercube (i.e., a lower-dimensional cube embedded in a higher-dimensional cube). One can go from the space of n-spheres to that of n-cubes using well-defined stereographic projections^14^. Such projections facilitate reduction of a (n + 1)- hypersurface to an n-hypersurface, provide a natural framework to capture multiple dimensions and hence also the structural representation of multidimensional stratified clinical trial data vis-à-vis ‘*master protocols*’ comprising multiple tumour types, multiple mutations and multiple drugs^16–18^. We propose that the design space of a master protocol is a hypersurface (hypersphere or hypercube) where Basket and Umbrella trial designs emerge as subspaces via projections of such hypersurfaces. In order to establish the geometric representation for master protocols, we consider the 3-cube (boundary of a 4-cube) which is an ordinary cube that can be thought of as a ‘Master Manifold’ (i.e., design space of a master protocol) with the three different dimensions representing (Fig. 2a) 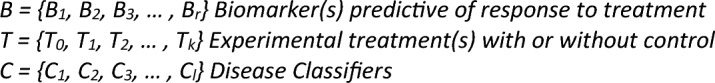 together with examples of dimensions B and C given for targeted cancer therapies (Fig. 2b).

The proposed master manifold can be cast for any therapeutic area. Given our focus of oncology, let us now illustrate how it can be cast for a targeted cancer therapy. The dimension T representing treatments, with or without the control arm T_0_, is carried over as is as a pre-requisite. The dimension B representing predictive biomarkers are populated with the DNA driver mutations while dimension C representing the disease classifiers are equated with the tissue-type or tumour-type. It is important to note that the axes B, T, and C of the master manifold are ‘nominal’ rather than continuous-valued, so it does not matter where along the axes the different components are placed as long as all elements of each dimension set are included. It would, therefore, not be right to assume that biomarker dimension B captures different levels of a particular biomarker; rather it captures a number of different biomarkers. By way of construction of the 3-cube, it is also assumed that the 3 dimensions chosen are mutually exclusive in that there is no overlap between any of the 3 axes B, T, and C. This makes perfect sense in the context of targeted cancer therapies since there will be little room for confusion among Treatments, Biomarkers, and Disease Classifiers.

**Figure 3:**
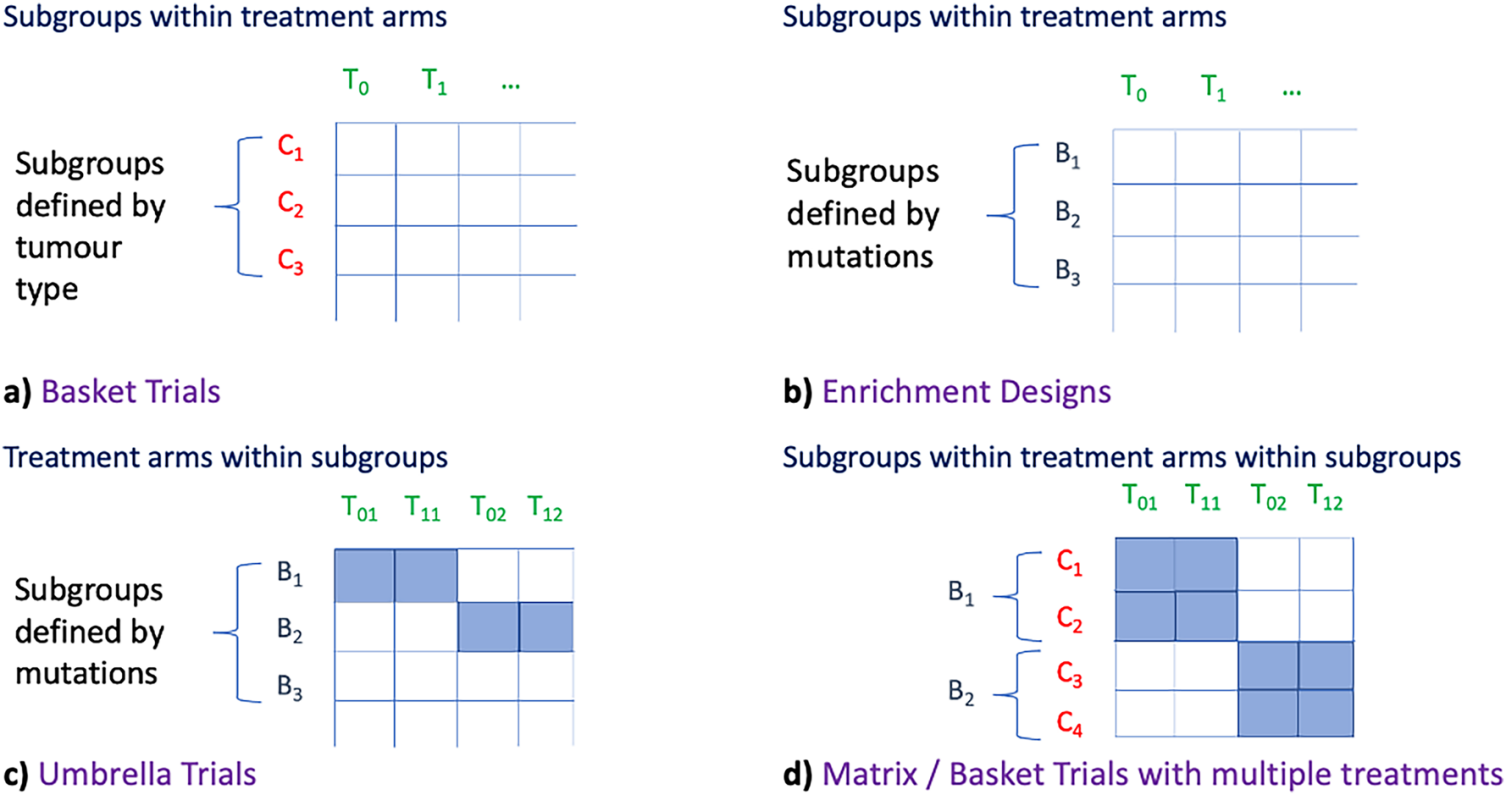
Projections yielding master protocol trial designs as subspaces.

The simplistic dimensional representation described above helps to visualise the design space of master protocols. Let us see how we can spot, or recover, or indeed create new basket and umbrella designs. Once we have identified the mutation(s) being targeted by the interventional drug’s mechanism of action, we can then fix the dimension B and taking projections on the 2-dimensional T,C plane yields the Basket trial designs (Fig. 3a). As expected, this design targets mutations present across different tumour-types. Next, if we focus on a particular tumour-type then we keep dimension C as fixed, take projections on the B,T plane that yields the Enrichment (Fig. 3b), Umbrella (Fig. 3c), and the more complex Matrix Trial designs (Fig. 3d), all obtained as embedded subspaces of the design space of master protocols. We recall that in Basket designs subgroups are defined by tumour type, whereas in Umbrella and Enrichment designs the subgroups are defined by mutations.

In the next section, we implement the framework of hypersurfaces to specific example of a basket trial design in oncology.

## Results

### Basket designs of Vemurafenib in melanoma and non-melanoma cancers with BRAF V600 mutation

**Figure 4:**
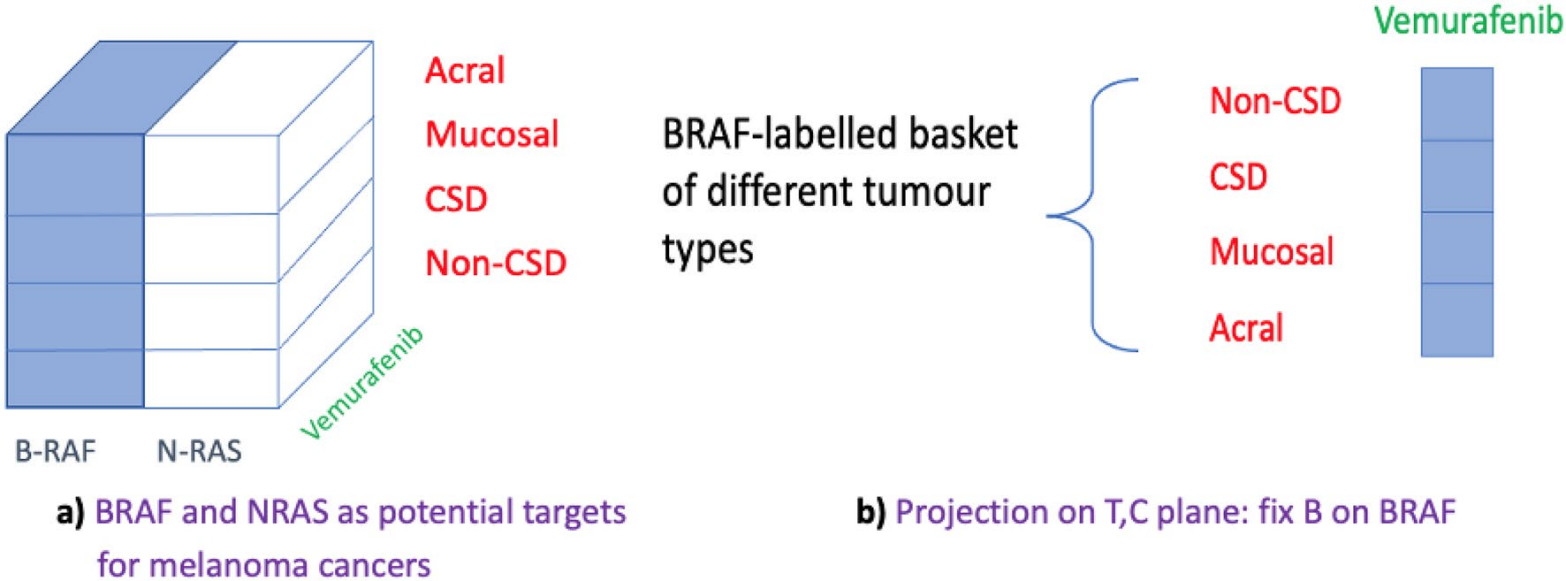
Significant association of BRAF with melanoma tumour types leading to basket design of Vemurafenib.

In^19^, distinct patterns of genetic alterations, namely the N-RAS and BRAF mutations, were studied in four groups of primary melanomas (Chronic Sun-induced Damage (CSD), Non-CSD, Mucosal, Acral) as these mutations were found in tissue samples with potential of being predictive of response to treatment. Analysis showed that mutations in BRAF were significantly associated with melanoma subtypes while N-RAS mutations did not exhibit any statistically significant association with the four types of melanomas. Given that the BRAF V600 mutation resulted in activation of down-stream signalling of mitogen-activated protein kinase, Vemurafenib was identified as a targeted drug that inhibits this kinase. This led to improved survival for the BRAF defined biomarker- positive subgroup in metastatic melanoma. We now summarise these findings geometrically using the master manifold (i.e., the 3-cube) defined above with the 3 dimensions:
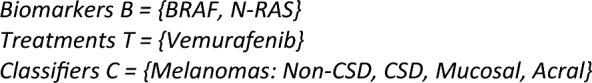
as shown in Fig. 4a. The shaded part of the hypersurface represents statistically significant association. This straightforward hypersurface then allows us to take a projection by fixing the dimension B on the significant BRAF mutation onto the 2D plane with the dimensions of Treatment and Classifiers (Fig. 4b). As one can see, this way of representation already yields a potential basket design of the then subsequent study of Vemurafenib in melanoma cancers.

**Figure 5:**
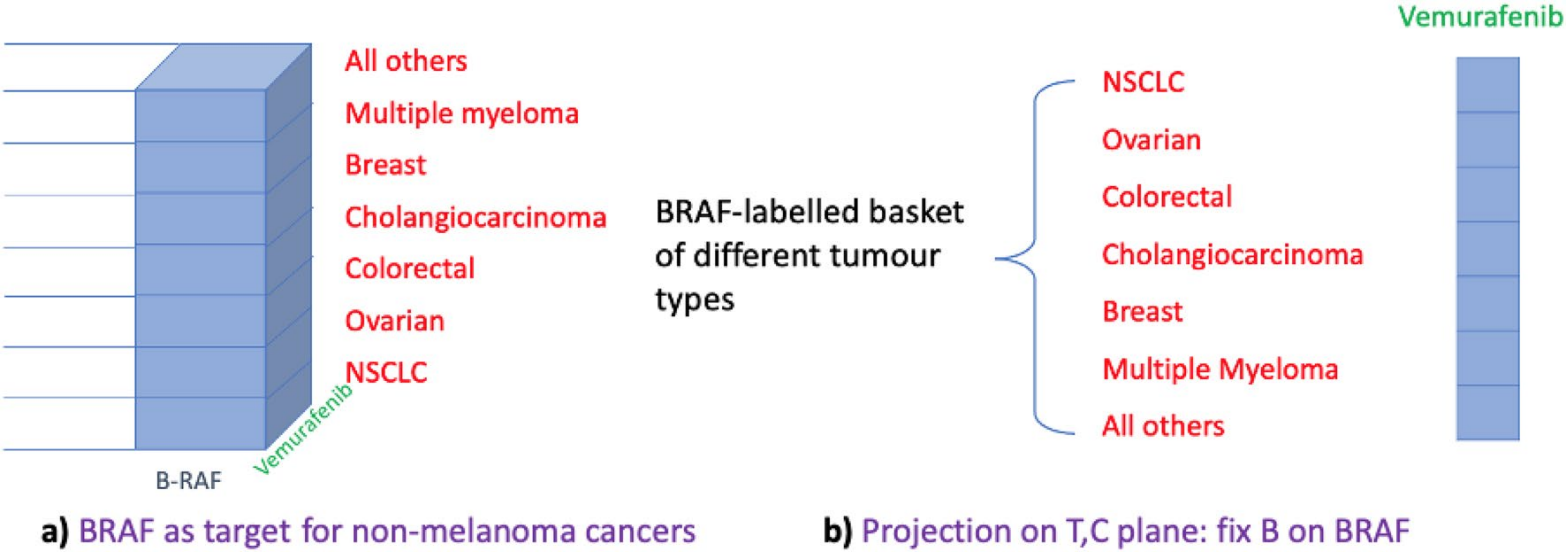
Original Vemurafenib basket trial design for BRAF-mutated nonmelanoma cancers.

Following the improved survival of BRAF-positive patients in melanoma cancers, the characterisation of the genetic landscape of tumour types identified the BRAF V600 mutations in many non-melanoma cancers too^20^ associated with poor survival outcomes. This then motivated one of the first known basket trials to study the efficacy of Vemurafenib in targeting certain BRAF V600 mutated nonmelanoma cancers^21^. The initial study design at the start of the trial differed from the study design published in^21^ since groupings of different tumour types were changed during the course of the trial. As such the published design also differs from the design following the adaptation. We now explain the design geometrically using the two different time points. At the start of the trial, the 3 dimensions of the master manifold (3-cube) were:
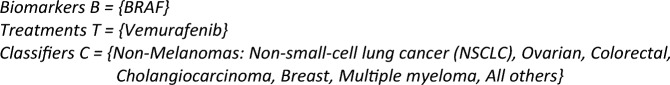
as shown in Fig. 5a. The ‘All others’ tumour types included cervical, brain tumour, head and neck, oesophageal and gastric, pancreatic, sarcoma, and unknown primary-type carcinoma. Again, taking a projection onto the 2D plane having fixed the biomarker BRAF yields the basket of different tumour types labelled by BRAF (Fig. 5b).

**Figure 6:**
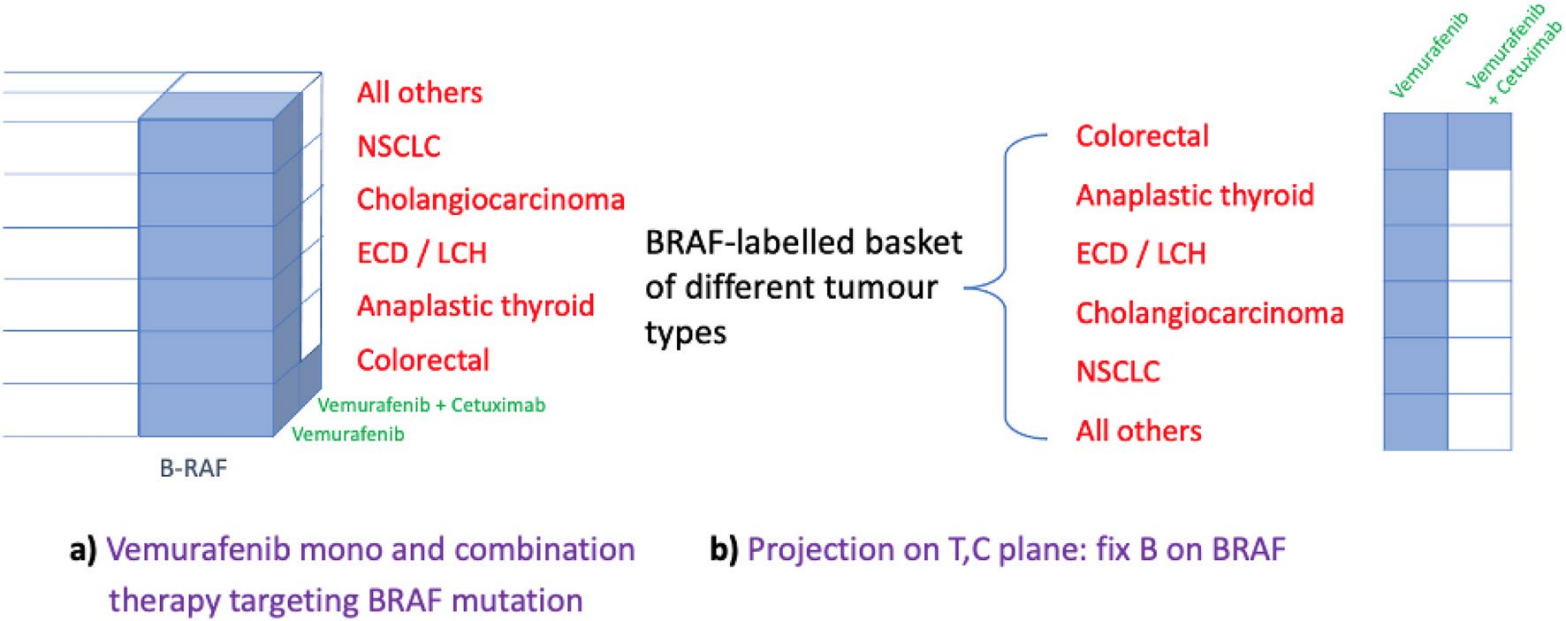
Vemurafenib basket trial design for BRAF-mutated nonmelanoma cancers following adaptation.

Adaptive platform design allowed for changes to be made during the trial. In particular, due to Vemurafenib resistance in colorectal cancer an anti-EGFR antibody, Cetuximab, needed to be combined for this tumour specific cohort. Further, due to early recruitment issues Breast cancers had to be included in the ‘All others’ subgroup while Ovarian and Multiple myeloma were dropped due to insufficient numbers. Instead, two new tumour types (disease classifiers) were added, namely the Erdheim-Chester Disease (ECD)/Langerhans-Cell Histiocytosis (LCH) and Anaplastic thyroid cancer. Any changes following adaptation are straightforward to tweak within the hypersurfaces framework by amending the dimension set of the 3-cube:
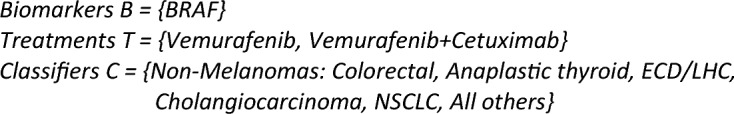
as shown in Fig. 6a. As before, projection from the biomarker on the 2D plane of Treatments and Classifiers yields the basket design of Vemurafenib (mono- and combination- therapy) targeting the BRAF mutation for the aforementioned non-melanoma cancers (Fig. 6b). This representation, therefore, is an accurate illustration of the tumour subtypes before and after adaptation when compared with the original study design figure published in^21^ which obscures the ‘All others’ cohort and incorrectly suggesting Ovarian and Multiple myeloma cancers as cohorts since these did not form part of the analysis.

Figures 4, 5 and 6 demonstrate the utility of the master manifold (3-cube) hypersurface framework to not only capture the statistically significant association geometrically but also relevant projections onto the 2D planes immediately discloses the basket trial design embedded in the manifold that would furnish useful visualisation tool for clinical investigators to plan a study. Similar to the above example of a basket trial design, the master manifold can be cast as an umbrella trial (e.g., FOCUS 4^4^ and Lung-MAP^5^), or indeed a more complex matrix trial^7,9^ involving multiple treatments, multiple mutations and/or multiple tumour types.

## Discussion

We have illustrated that the trial designs of basket, umbrella, and master protocols can be represented geometrically via hypersurfaces. This was plausible because master protocols have an underlying dataset that is inherently high-dimensional. In order to draw a hypothesis-driven inference from such high-dimensional genetic dataset, one risks missing out on the shape and structure of the overall data. Just as in a standard clinical trial, ‘small’ datasets are analysed using summary statistics to get an impression of the data, summarising high-dimensional data using tables and descriptive statistics may not serve to gain a deeper understanding of the data. Hence, appropriate visualisation techniques are required for exploratory analysis of high-dimensional biomedical data which becomes even more challenging when integrating different types of data e.g., clinical, gene expression etc. Geometry, being intrinsically a visual branch of mathematical science can aid the construction of such exploratory analysis that could not only help discover potential subgroups sharing a specific pattern of biomarker measurements but also provide a better understanding of the design of master protocols as well as guide supervised or unsupervised analysis.

In our novel geometric construction of the Master Manifold, we have shown how the proposed methods can be applied with real data by way of assigning each hypersurface dimension with disease classifier, predictive biomarkers, and treatment arms. This has been further elucidated by fitting in a concrete example from targeted cancer therapy with tumour-types and mutations. In future work, subsets of high-dimensional cancer data will be used to generate real-world hypersurfaces whose visualisation in terms of the shape and structure will guide trialists and oncologists which parts of the surface should be focus areas to help achieve objectives of efficacy studies, similar to how contour plots of the dual drug or dose escalation models help investigators focus on Phase 1 studies to achieve maximum tolerated doses or dose limiting toxicities.

Focus areas for future research emanating from our approach may include representing exploratory analysis using hypersurfaces to visualise patterns of biomarker data recorded as well as missing^22^, visualise tumour-agnostic significant associations^23^ to help identify underlying potential trial designs, and integrate multimodal data^24^ along the multiple dimensions of a hypersurface to gain a holistic understanding of seemingly disparate data types contributing to desired disease outcomes.

The geometric representation of master protocols in terms of 3-dimensional hypersurfaces established here paves the way for generalisation to 4-dimensional and potentially higher-dimensional innovative trial designs for cancer therapeutics where the interest is in developing multi-omic drugs. Therefore, in addition to the drug targeting the disease classifier and biomarker or mutation, additional biological targets would be selected from a panel of transcriptomics, proteomics and other single-cell multi-omics that can help design more effective personalised treatments and combination therapies that better incorporate the tumour microenvironment.

## Data Availability

No datasets were generated or analysed during the current study.
